# Mitoferrin, Cellular and Mitochondrial Iron Homeostasis

**DOI:** 10.3390/cells11213464

**Published:** 2022-11-02

**Authors:** Md Yousuf Ali, Claudia R. Oliva, Susanne Flor, Corinne E. Griguer

**Affiliations:** 1 Free Radical & Radiation Biology Program, Department of Radiation Oncology, The University of Iowa, Iowa City, IA 52242, USA; 2 Interdisciplinary Graduate Program in Human Toxicology, The University of Iowa, Iowa City, IA 52242, USA

**Keywords:** iron, mitoferrin, mitochondria, cancer, erythropoiesis, oxidative stress

## Abstract

Iron is essential for many cellular processes, but cellular iron homeostasis must be maintained to ensure the balance of cellular signaling processes and prevent disease. Iron transport in and out of the cell and cellular organelles is crucial in this regard. The transport of iron into the mitochondria is particularly important, as heme and the majority of iron-sulfur clusters are synthesized in this organelle. Iron is also required for the production of mitochondrial complexes that contain these iron-sulfur clusters and heme. As the principal iron importers in the mitochondria of human cells, the mitoferrins have emerged as critical regulators of cytosolic and mitochondrial iron homeostasis. Here, we review the discovery and structure of the mitoferrins, as well as the significance of these proteins in maintaining cytosolic and mitochondrial iron homeostasis for the prevention of cancer and many other diseases.

## 1. Introduction

Iron uptake by cells is essential to fulfill cellular iron requirements for many metabolic processes, including electron transport, nucleotide synthesis, oxygen transport, and redox reactions [1]. Mitochondria consume a large portion of the intracellular iron to synthesize iron-sulfur (Fe/S) clusters and heme necessary to support these and other vital cell processes [2,3,4,5]. The high redox potential of iron facilitates the reactions necessary for these processes, but it can also have pathologic consequences, including the increased mitochondrial production of reactive oxygen species (ROS) and even cell death, when iron homeostasis is disrupted. Therefore, precise cellular and mitochondrial iron uptake is essential to maintain iron homeostasis and health [6,7].

**Figure 1:**
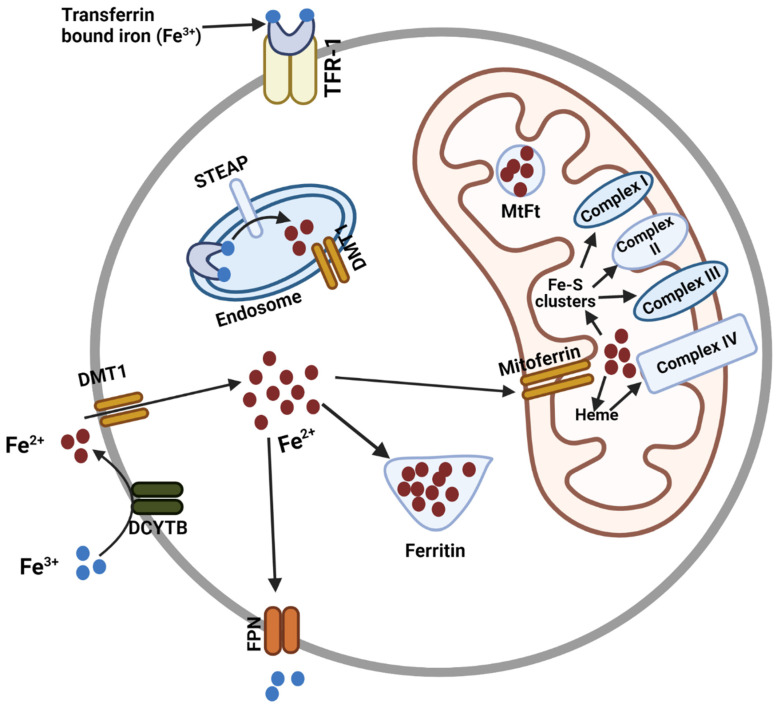
Overview of cellular and mitochondrial iron homeostasis. Tf-bound iron (Fe^3+^) is internalized into cytosol through TFR-1. Fe^3+^ is then converted to Fe^2+^ by STEAP in endosomes. Endosomal Fe^2+^ is then released into the cytosol thorough DMT1. A small portion of iron that is not bound to Tf can be converted to Fe^2+^ by duodenal cytochrome B (DCYTB) and incorporated into cytosol by DMT1. These cytosolic Fe^2+^ can then be stored in ferritin, exported out of the cell by ferroportin (FPN), or transported to the mitochondria by mitoferrin. In the mitochondria, iron is used for the generation of iron-sulfur clusters or heme synthesis. The iron sulfur (Fe-S) clusters and heme are used in different proteins and enzymes in the mitochondria. Fe-S clusters and heme can be exported to the cytosol for other cellular utilization. Iron can be stored in mitochondrial ferritin (MtFt) as well. Created with BioRender (Science Suite Inc., Toronto, ON, Canada).

Organisms ranging from prokaryotes to plants and mammals have developed sophisticated, high-affinity uptake mechanisms with corresponding receptors to regulate the incorporation of iron [8,9,10,11]. Transferrin (Tf), a protein with high affinity for iron, tightly binds extracellular ferric (Fe^3+^) iron in the circulating plasma. The binding of the Tf(Fe^3+^) complex to the transferrin receptor-1 (TfR1) on cells then facilitates receptor-mediated endocytosis of the Tf-TfR1 complex. When the internalization of iron is necessary to meet cellular demand, the expression of the transferrin receptor-1 (TfR1) is upregulated on the cell surface to bind and endocytose the Tf(Fe^3+^) complex. The acidic pH within the endosomes reduces the affinity of Tf for Fe^3+^, triggering the release of Fe^3+^ from the complex [12] and the subsequent reduction of Fe^3+^ to Fe^2+^ by the metalloreductase, Steap3. Divalent metal transporter 1 (DMT1) is known to transport reduced iron out of endosomes [13,14]. A small portion of iron is also incorporated into the cell by DMT1. Non-transferrin-bound iron is reduced to Fe^2+^ by ferrireductases at the cell surface and transported into cytosol by DMT1 [15]. The majority of the reduced Fe^2+^ is then shuttled into the mitochondria by mitoferrins and used for the mitochondrial biogenesis of heme and Fe/S clusters [1,5] (Figure 1).

Two mitoferrins, mitoferrin-1 (encoded by the solute carrier family 25-member 37 gene, *SLC25A37*) and mitoferrin-2 (encoded by *SLC25A28*), have been identified in humans, with homologs identified in many other eukaryotes as well. As the mitoferrins control the crucial balance of iron in the cytoplasm and mitochondria, it is not surprising that the dysregulation of these proteins has been implicated in a variety of human diseases and disorders. In this review, we discuss the discovery, structure, and regulation of the mitoferrins, particularly in vertebrates, as well as the evolution of these proteins through different species. Furthermore, the association of the mitoferrins with various developmental processes and disease development will also be discussed.

## 2. Discovery of Mitoferrins

The first mitoferrin homologs were discovered by Wiesenberger et al. in the inner mitochondrial membrane of *Saccharomyces cerevisiae* (yeast) in 1991 [16]. These proteins, referred to as mitochondrial RNA splicing 3 [Mrs3] and Mrs4, were found to suppress mitochondrial intron splicing defects. However, an analysis of the primary and secondary structures revealed Mrs3 and Mrs4 to be members of the mitochondrial solute carrier protein family, leading the authors to conclude that Mrs3 and Mrs4 control mitochondrial RNA splicing by regulating mitochondrial solute concentrations. Although it was soon reported that Mrs3 or Mrs4 could be substituted for Mrs2 in the mitochondrial import of magnesium in yeast [17], the role for these proteins in iron transport was not suggested for nearly a decade, when Rutherford et al. found that *MRS4* is co-induced with other iron-uptake genes in yeast with a mutation in the iron-responsive transcriptional activator Aft2p [18]. Soon after this publication, it was demonstrated that the deletion of *MRS3* and *MRS4* suppresses mitochondrial iron accumulation in a frataxin-deficient yeast strain [19] and that the deletion of *MRS3* and *MRS4* impairs iron metabolism and mitochondrial Fe-S cluster biogenesis in yeast when cytosolic iron is limited [19,20,21,22].

In 2006, Shaw et al. [23] identified mitoferrin-1, a member of the vertebrate mitochondrial solute carrier family of proteins and a homolog of Mrs3/4, as the mutated protein responsible for the profound anemia and erythroid maturation arrest in the *frascati* zebrafish (*Danio rerio*) mutant. Their additional studies in mice confirmed that mitoferrin-1 functions as an essential mitochondrial iron importer in developing mammalian erythrocytes, specifically [23]. Although mitoferrin-1 was found to be expressed predominately in erythroid tissue, with only minimal expression in other cells, the paralog mitoferrin-2, also identified in this study, was found to be ubiquitously expressed in zebrafish and mice. Interestingly, the introduction of either mammalian mitoferrin rescued the mitochondrial iron needs of yeast deficient in Mrs3 and Mrs4, yet mitoferrin-2 could not rescue the anemic phenotype of *frascati*. On the basis of these results, the authors proposed that mitoferrin-2 mediates the mitochondrial import of iron required for the synthesis of heme-containing proteins and Fe/S cluster assembly in non-erythroid cells, whereas mitoferrin-1 is essential to meet the higher demand for mitochondrial iron to support heme production in erythroid cells [23]. Subsequent in vitro studies by the same group validated this hypothesis and characterized the stage-specific regulation of mitoferrin-1 in developing erythroid cells [24], and their studies in knockout (KO) mice revealed that the absence of mitoferrin-1 leads to embryonic lethality at the time of erythrocyte development [25]. Overall, this work indicates that the mitoferrins function as redundant mitochondrial iron importers in non-erythroid cells, but mitoferrin 1 is essential when the demand for mitochondrial iron is high.

## 3. Mitoferrin Structure and Conservation of Structure in Different Species

The human genes encoding mitoferrin-1 and mitoferrin-2 are located in two different chromosomes and vary in transcript, exon number, and protein length, as shown in Table 1.

**Figure 2:**
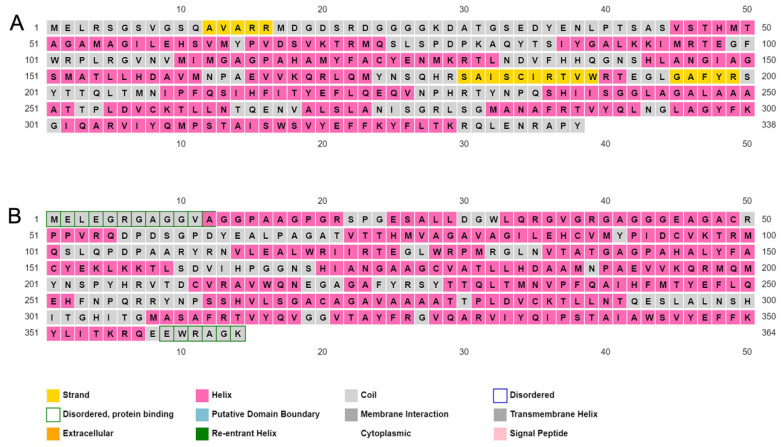
Predicted secondary structures of human mitoferrin-1 (**A**) and mitoferrin-2 (**B**). The protein sequences were analyzed using PSIPRED to identify protein secondary structure. The predicted alpha-helices, beta-strands, coiled coils, and disordered regions are indicated by unique colors, as detailed in the legend below each sequence.

Consistent with these differences, an analysis of the secondary structures of human mitoferrin-1 and mitoferrin-2 using the PSIPRED protein analysis tool [26,27] predicted differences in the secondary structure of the two proteins as well (Figure 2). Specifically, the analysis predicted a beta-strand (yellow) formation site in mitoferrin-1, but not in mitoferrin-2, and unique disordered protein binding regions in the N- and C-terminal regions of mitoferrin 2 (green outlines), but not in those of mitoferrin 1. Although alpha-helices (pink) were predicted in mitoferrin-1 and in mitoferrin-2, the regions of helical formation differ between the two proteins. No cytoplasmic or extracellular domains were predicted for either mitoferrin.

**Figure 3:**
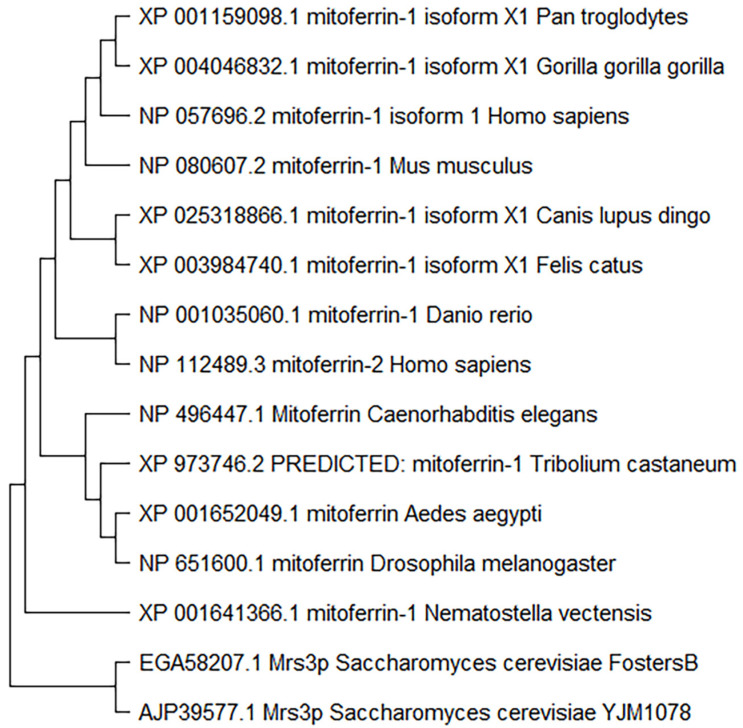
Maximum likelihood phylogenetic tree of mitoferrin-1 protein sequences. The length of the branches indicates evolutionary time. The protein sequences were selected by BLASTP and aligned using MUSCLE alignment. The maximum likelihood phylogenetic tree was then constructed using MEGAX (v11).

**Figure 4:**
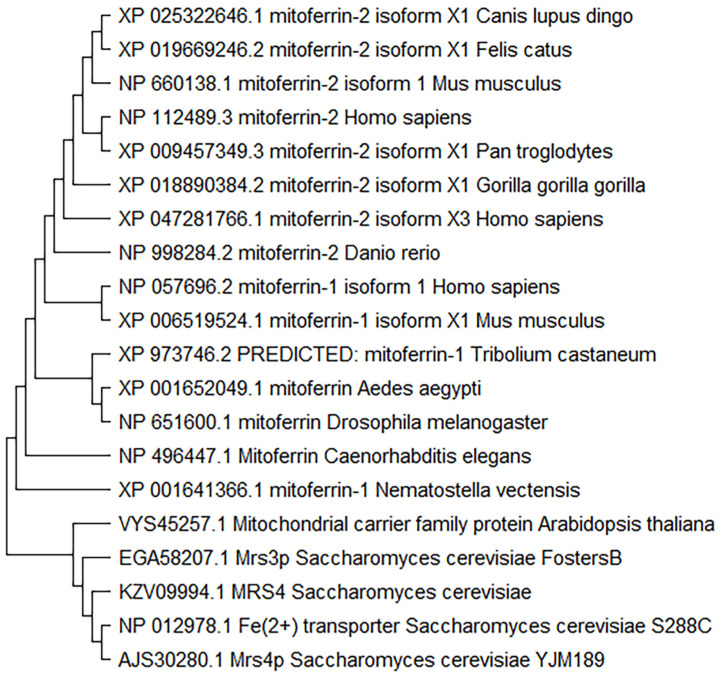
Maximum likelihood phylogenetic tree of Mitoferrin-2 protein sequences. The length of the branches indicates evolutionary time. The protein sequences were selected by BLASTP and aligned using MUSCLE alignment. The maximum likelihood phylogenetic tree was then constructed using MEGAX (v11).

The data showing that mitoferrins of one species can compensate for the homologs in another species indicate that the function of the respective genes is highly conserved [23]. However, a thorough analysis of the diversity of the mitoferrins across species and phyla has not been published. Therefore, we performed a detailed analysis of phylogenetic relationships among mitoferrin-1 and mitoferrin-2, examining the homologs from several eukaryotes and species from different phyla. The maximum-likelihood phylogenetic analysis of the mitoferrins showed *Saccharomyces cerevisiae* as an outgroup for both mitoferrin-1 and mitoferrin-2 (Figure 2 and Figure 3). The placement of yeast as a more distant cluster indicates a clear segregation between unicellular and multicellular eukaryotes. Among the mitoferrin-1 homologs, the homolog closest in sequence to that in the outgroup was from a nematode (*Nematostella vectensis*) (Figure 3), whereas among the mitoferrin-2 homologs, the homolog closest in sequence to that in the outgroup was from a plant species (*Arabidopsis thaliana*) (Figure 4).

Although the homologs of mitoferrin-1 and mitoferrin-2 displayed a similar phylogenetic distribution among different species, the mitoferrin homolog in *Arabidopsis thaliana* only appeared in the evolutionary distribution of mitoferrin-2. Interestingly, human mitoferrin-1 was more closely related to mitoferrin-1 of other mammals than to human mitoferrin-2 (Figure 3), which was in a sister group with an isoform of *Danio rerio* mitoferrin. Mitoferrin homologs in insects were found in the same subgroups as those of *Caenorhabditis elegans* (nematode). Finally, human mitoferrins were sub-grouped with mitoferrins of other mammalian species. Despite these differences in the branching among phyla and species, it appears from the length of the branches that the mitoferrin proteins are well conserved among different species.

The mechanism of mitoferrin-mediated iron delivery to the mitochondria is not yet clearly understood. However, Brazolotto et al. identified three highly conserved histidine residues in the yeast homologues of mitoferrin-1 and mitoferrin-2 that are required for iron to glide across the mitochondria [28]. Divalent metal transporter 1 (DMT1) also has similar histidine residues that contribute to the iron carrier function [29]. Characterization of the iron-transporting function of the mitoferrins, however, was delayed until Christenson et al. developed techniques to overcome the complications associated with iron-mediated redox reactions in liposomal transport assays. Their analyses confirmed that mitoferrin-1 binds Fe^2+^ with high affinity (~10^2^ µM) and, although the activity of mitoferrin is not highly dependent on pH, its iron transport activity is faster when the pH outside the liposomes is alkaline, which is physiologically consistent with the mitochondrial pH. Furthermore, three amino acid residues, histidine, cysteine, and methionine, are conserved among mitoferrin-1 and its orthologs. These three amino acids are critical for the transport activity of iron [30]. The conservation of the three key amino acids involved in iron transport shows the importance of mitoferrin in transporting iron to the mitochondria in different species.

## 4. Regulation of Mitoferrin Expression

Differences in transcriptional and post-transcriptional regulation likely underly the cell-specific expression and function of mitoferrin-1 and mitoferrin-2 in the species with both paralogs. Table 2 summarizes the known factors and regulators of mitoferrin reported in different studies. In addition to identifying the function of these mitoferrins in vertebrates, the study by Shaw et al. revealed that the expression of the mitoferrin-encoding gene is deficient in *gata1* mutant zebrafish, providing the first evidence that the master erythroid transcription factor GATA-1 regulates the stage- and tissue-specific expression of mitoferrin-1 [23]. Using genome-wide predictions by chromatin occupancy and in vivo reporter gene expression assays, the same group later confirmed that GATA-1 stage-dependently displaces GATA-2 binding at two cis-regulatory elements upstream of mitoferrin-1 to induce mitoferrin-1 transcription during erythroid maturation [31]. Interestingly, the transcript expression of both mitoferrins was detected in the central nervous system (CNS) of zebrafish embryos. Moreover, the cis-regulatory region to which GATA-1 binds upstream of mitoferrin-1 drove GFP expression in the CNS as well as the heart, suggesting other GATA factors could regulate the CNS expression of mitoferrin-1.

Finally, this group identified binding motifs for the transcription factors involved in muscle differentiation, namely MyoD, myogenin, and Myf-5, in the mitoferrin-1 cis-regulatory module, although the relevance of these motifs was not further evaluated [31].

Iron regulatory protein 1 (IRP1) has also been implicated in the transcriptional regulation of mitoferrin-1 expression. Specifically, Zhang et al. recently reported that the glycolytic enzyme α-enolase 1 (ENO-1) inhibits the expression of mitoferrin-1 by reducing *IRP1* mRNA expression, which in turn prevents the transcription of *SLC25A37* by CREB, ultimately reducing ferroptosis in hepatocellular carcinoma cells [32]. Seemingly in contrast, however, an analysis of RNA-sequencing data from TCGA and GTEx data revealed that the expression of *ENO-1* (and others) positively correlated with the expression of *SLC25A37* in sarcoma patients, but the molecular mechanisms underlying this correlation were not directly examined [33]. These disparate findings suggest that the factors regulating mitosferrin expression differ by cell type and pathogenic state and caution against universal conclusions.

In a study designed to characterize the function of enhancers as determinants of cell identity, Huang et al. [34] identified a GATA-1-binding enhancer cluster defined as an erythroid-specific super-enhancer upstream of *SLC25A37* in human and mouse erythroid cells. CRISPR/Cas9-mediated genomic editing revealed developmental stage-specific requirements for the constituent enhancers in *SLC25A37* mRNA expression during the differentiation of G1ER murine erythroid cells. These findings shed additional light on the transcriptional regulation of mitoferrin-1 expression, even though the effects on mitoferrin-1 function were not examined.

Posttranscriptional mechanisms have been shown to regulate the expression of mitoferrin-1 and mitoferrin-2 as well. Using affinity purification and mass spectrometric analysis, Chen et al. identified that mitochondrial inner membrane ATP-binding cassette transporter ABCB10, which is also regulated by GATA-1 in erythroid cells [35], directly binds to mitoferrin-1, stabilizing and increasing the half-life of the mitoferrin-1 in differentiated mouse erythroleukemia (MEL) cells. This binding ultimately led to the increased incorporation of iron into the mitochondria. In contrast, ABCB10 did not interact with mitoferrin-2 at any point during the maturation of these cells [36]. Additional studies revealed that mitoferrin-1 and ABCB10 also form a transient oligomeric complex with the final enzyme of the heme synthesis pathway, ferrochelatase, during erythroid differentiation of MEL cells to facilitate the transfer of iron to ferrochelatase and, thus, support heme synthesis [37].

A transcriptome-wide analysis of MIA pancreatic cancer cells stably overexpressing the RNA m^6^A demethylase ALKBH5 revealed that ALKBH5 regulated the methylation of *SLC25A28*, enhancing the RNA stability, as well as the methylation and alternative splicing of *SLC25A37*, leading to upregulation of the respective mitoferrin proteins. RIP-qPCR analysis confirmed a direct interaction between ALKBH5 and *SLC25A28*, and *SLC25A37* RNA [38]. However, the mitoferrin proteins were upregulated in PDAC cells from pancreatic cancer patients despite the decreased expression of ALKBH5 [38], again highlighting the likely complexity of mitoferrin regulation in vivo, particularly in cancer cells. This study is fairly unique, as few other studies have identified direct regulators of mitoferrin-2. Among these, a study with mouse aortic endothelial cells revealed that the direct binding of mitoferrin-2 by 14-3-3e prevents the ubiquitination and proteosomal degradation of mitoferrin-2 in, which led to mitochondrial iron overload [39]. Additionally, target gene prediction and expression analysis identified *SLC25A28* as a target of miR-132 in mouse and human primary pancreatic islet cells [40]. The silencing of miR-132 in mice or isolated islets improved insulin secretion, but the specific contribution of mitoferrin-2 in this process was not determined.

miRNA regulation of mitoferrin-1 has been documented as well. RNA sequencing revealed the downregulation of *SLC25A37* mRNA and protein expression, as well as mitochondrial dysfunction and cell death in human rhabdomyosarcoma RD cells overexpressing miR-7. Luciferase reporter assays confirmed that miR-7 directly targets the *SLC25A37* 3’UTR, and siRNA-mediated silencing of *SLC25A37* alone induced mitochondria dysfunction and cell death in RD cells [41]. Furthermore, Lenkala et al. [42] found that the overexpression of miR-22 downregulates the expression of *SLC25A37* in ovarian cancer cells. Although the miRBase miRanda algorithm did identify *SLC25A37* as a predicted target of miR-22 in a subset of HapMap lymphoblastoid cell lines, the direct binding of *SLC25A37* by miR-22 was not examined [42].

Indeed, without determining the specific underlying mechanisms, several studies have identified other regulators of mitoferrin expression. A recent study in differentiating murine G1E-ER4 cells, a model of early erythrocyte development, revealed that the knockdown of ABCB7 increases the mitochondrial expression of mitoferrin-1, which correlated with an increase in mitochondrial iron levels. The increased expression of mitoferrin-1 required the expression of iron regulatory protein 2 (IRP2), but no direct interactions with mitoferrin-1 were reported [43]. Genome-wide transcription by high-throughput transcriptome sequencing revealed the downregulation of *slc25A37* in zebrafish deficient in the large ribosomal subunit protein Rpl11, a model of Diamond-Blackfan anemia. Furthermore, pharmacologic inhibition of ERK led to upregulation of mitoferrin-1 mRNA and iron incorporation into heme, whereas blocking p38 MAPK appeared to have the opposite effect in MEL cells induced to express the erythroid phenotype [44]. As several studies have shown the involvement of the MAPK and PI3K/Akt pathways in erythroid differentiation [45], this is perhaps unsurprising.

Suggesting the regulation of *SLC25A37* by the splicing factor SF3B1, a splice variant of *SLC25A37* was detected in the bone marrow cells of patients with myelodysplastic syndrome (MDS) and a mutation SF3B1, the gene most often mutated in MDS [46]. Ultrastructural analysis using transmission electron microscopy revealed that the mitochondrial iron content in these cells was far higher than that in the cells of patients without the SF3B1 mutation. Similarly, a variant of mitoferrin-1 containing an insert of intron 2 with a stop codon was detected in blood leukocytes and liver tissue from patients with erythropoietic protoporphyria [47]. In fact, the mitoferrin-1 splice variants detected in these two studies are the same as those detected in a study of ALKBH5 in pancreatic cancer cells [38].

Using mouse models and human cell lines, Li et al. [48] found that the PINK1-PARK2 pathway normally associated with mitophagy also controls the protein expression of mitoferrin-1 and mitoferrin-2 and mitochondrial loading in pancreatic cancer cells via a mechanism requiring the ATG5-dependent autophagy pathway rather than mitophagy. Interestingly, a subsequent study showed that *Slc25a28* expression was downregulated in *Pink*−/− mouse embryonic fibroblasts upon iron depletion, whereas the expression of *Slc25a37* did not change. In contrast, *Slc25a37* was upregulated upon iron overload in the same cells. Finally, stable overexpression of heme oxygenase-1 (HO-1) led to an increase in mitoferrin-2 mRNA expression and in mitochondrial iron content in HEK293 cells, indicating a role of HO-1 in mitoferrin-2 expression [49].

**Table 2:** Summary of factors and regulators regulating mitoferrin expression and iron transport activity.

Factors or Regulators of Mitoferrin	Effect in Mitoferrin Expression or Activity	References
1. GATA-1	Displaces GATA-2 from two cis-regulatory elements upstream of mitoferrin-1 to induce mitoferrin-1 transcription during erythroid maturation	[23,31]
2. ENO-1	Inhibit the transcription of mitoferrin-1 by reducing IRP1 mRNA expression in head and neck cancer cells	[32]
3. ABCB10	Stabilizes mitoferrin-1 by directly binding to it	[36]
4. ALKBH5	Regulate the methylation of mitoferrin-2 and enhances RNA stability of mitoferrin-2 in pancreatic ductal carcinoma cells.	[38]
5. miR-7	Directly target 3’-UTR of mitoferrin-1 and silences it in rhabdomyosarcoma	[41]
6. PINK1 and PARK2	Regulates the expression of mitoferrin-1 and mitoferrin-2 using ATG5 dependent autophagy pathway	[48]
7. pH	Faster iron transport activity at alkaline pH	[30]

All these studies reporting the regulation of mitoferrin represent a complex mechanism involved in maintenance of this critical mitochondrial iron transporter. It is not distinctively clear if mitoferrin is regulated at the transcriptional, translational, or post-translational level. Moreover, it is still unknown how the level of cellular and mitochondrial iron dictates the expression of mitoferrin in different physiological conditions. Furthermore, it is also not clearly understood how mitoferrin expression and iron transport activity is controlled in a limited iron condition in the context of iron utilization, storage, and export. Therefore, more studies are needed to better understand the regulation of mitoferrin in the context of overall cellular and mitochondrial iron homeostasis.

## 5. Importance of Mitoferrin in Normal Physiology and Disease Development

Considering the established connections between mitochondrial dysfunction and disrupted iron homeostasis and a variety of pathologic conditions, including atherosclerosis, type 2 diabetes, neurodegenerative diseases such as Parkinson disease, Alzheimer disease, and amyotrophic lateral sclerosis, and cancer progression and resistance to treatment among others [50,51,52,53,54,55,56,57], it is not surprising that research is uncovering similar connections to the absence or dysfunction of mitoferrins. We will focus here on the consequences associated with dysregulated expression of the mitoferrins in animals (Table 3), but it is certainly worth mentioning that the dysregulation of the single mitoferrin homolog expressed has also been linked to abnormal growth and development in plants [58,59,60,61].

As already discussed, eukaryotic mitoferrin-1 was originally found to be expressed primarily in erythroid tissue and essential for erythropoiesis and embryonic survival, with mutations in this gene linked to severe anemia. Therefore, it is not surprising that dysregulated expression of mitoferrin 1 was detected in the hematopoietic stem cells of patients with myelodysplastic syndrome [46,62]. However, despite the relatively low expression detected in non-erythroid tissue, this expression appears to be physiologically important. For example, hepatocyte-specific loss of mitoferrin-1 has been associated with defective iron homeostasis leading to protoporphyria and hepatotoxicity in mice under conditions of increased heme synthesis [25]. Moreover, mitoferrin-1 deficiency caused by the insert of a stop codon in intron 2 was identified in the lymphoblasts of patients with erythropoietic protoporphyria who did not have mutations in FECH, the enzyme that is most often associated with this disorder. Because mitoferrin-1 normally functions in a complex with FECH and ABCB10, it was proposed that the deficiency of mitoferrin-1 inhibits FECH function in these patients [47].

Perhaps providing an explanation for the high prevalence (22%) of anemia among patients with major depressive disorder (MDD) [63], a meta-analysis of GWAS data strongly suggested that SCL25A37 mitoferrin-1 is also a risk gene for MDD [64] and is downregulated in the hippocampus and peripheral blood of patients with MDD. Evidence further suggests that mitoferrin-1 is required for proper brain energy metabolism and hippocampus-dependent memory as well. For example, the neuron-specific knockout of mitoferrin-1 in mice is associated with diminished neuronal energy metabolism and impairments in spatial learning and memory [65]. In drosophila, the overexpression of the one mitoferrin homologue improved the symptoms of Parkinson disease by rescuing the impaired mitochondrial function caused by a parkin1 (PINK1) loss of function mutation [66].

Emphasizing the need for the precise regulation of mitochondrial iron, increased expression of mitoferrin-1 has also been linked to neuronal disorders such as Friedreic ataxia (FRDA), a disease characterized by neurodegeneration and cardiomyopathy caused by the loss of function of frataxin, a protein involved in generation of Fe/S clusters in mitochondria. Upregulated expression of mitoferrin was detected in a *Drosophila* model of FRDA, and the genetic suppression of mitoferrin counteracted the molecular and physiological effects of frataxin deficiency, although it decreased the life span of the flies [67,68]. The downregulation of mitoferrin-1 also slowed disease progression by altering mitochondrial iron metabolism and the production of reactive oxygen species in a *C. elegans* model of Alzheimer disease [69]. Increased expression of mitoferrin-1 was also associated with an increase in the labile iron pool and mitochondrial damage observed with aging in adult skeletal muscle [70].

In contrast to mitoferrin-1, mitoferrin-2 was originally found to be ubiquitously expressed and unable to compensate for mitoferrin-1 depletion in the context of upregulated heme synthesis. Research since the discovery of mitoferrin-2 has revealed that global knockout in mice does not cause obvious hematologic dysfunction or affect viability but instead reduces sperm number and motility in males, thereby diminishing fertility [71]. Recessive male sterility resulting from decreased spermatogenesis was also reported in Drosophila in which the one mitoferrin gene was deleted [72], suggesting the universal role of mitoferrin in fertility.

In mice, global loss of mitoferrin-2 was also associated with changes in the mitochondrial levels of several minerals, including iron, copper, cobalt, and zinc, but this only occurred with a low-iron diet [71]. Hepatocyte-specific knockout of mitoferrin-1 in the mitoferrin-2 knockout animals did reduce mitochondrial iron levels in mice on a normal diet, however, and this effect was also enhanced in mice fed a low-iron diet, reminiscent of the results in Mrs3/4-depleted yeast. Furthermore, loss of both mitoferrins in hepatocytes impaired liver regeneration in adult mice, possibly as a result of diminished amino acid production. Similarly, primary bone marrow-derived macrophages or immortalized fibroblasts derived from the mitoferrin-2-knockout mice and treated to knockout mitoferrin-1 did not proliferate in vitro, likely due to the accompanying reduction in mitochondrial oxidative phosphorylation proteins detected in the fibroblasts. Finally, knockdown of both mitofferin-1 and mitoferrin-2 led to mitochondrial dysfunction, impaired insulin sensitivity, and suppressed adipogenic differentiation in 3T3-L1 preadipocytes [73]. Although mitochondrial dysfunction has been linked to metabolic disease, to our knowledge, mitoferrin dysregulation has not yet been reported in patients with diabetes or obesity.

As with mitoferrin-1, an aberrant increase in mitoferrin-2 expression may also have pathologic consequences. Upregulated expression of mitoferrin-2 was detected in the aortic endothelial cells of a mouse model of atherosclerosis, and knockdown of this protein reduced endothelial dysfunction by decreasing the mitochondrial iron level [39]. In addition, mitoferrin-2 accumulation was detected in the brains of mice and humans with Huntington disease and correlated with a decrease in frataxin expression and mitochondrial bioenergetic dysfunction due to iron overload in the mitochondria of brain cells [74].

Jump-starting the ongoing interest in the intriguing role of mitoferrins in the development, progression, and treatment resistance of cancer cells, in 2013, Hung et al. discovered that mitoferrin-2 is upregulated in head and neck cancer cells that are sensitive to radiation and actually confers this sensitivity by increasing the mitochondrial uptake of iron and, thus, mitochondrial dysfunction [75]. Conversely, Wang et al. reported that mitoferrin-2 expression is required for the ROS-mediated apoptosis and cytotoxicity induced by arsenic-trioxide treatment in glioma cells [76]. A recent study by Tomita et al. [77] similarly revealed that mitoferrin-1 is required for treatment efficacy in HeLa and oral squamous cell, as the silencing of mitoferrin-1 led to radioresistance in these cultured cells. Moreover, the induction of radioresistance by exposure to fractionated radiation in these cells associated with reductions in mitoferrin-1 expression and mitochondrial iron concentration in these cells, providing clinically relevant evidence that the aberrant depletion of mitoferrin-1 drives the acquisition of radioresistance in at least some cancer cells. Evidence from in vitro and in vivo studies has also suggested that the downregulation of mitoferrin-1 detected in hepatocellular carcinoma cells drives cell survival and tumor growth by minimizing mitochondrial iron-induced ferroptosis [32].

In osteosarcoma cell lines, Ni et al. [33] demonstrated that mitochondrial ROS production is critically involved in the iron-mediated induction of the Warburg effect, and this effect is controlled by the expression of mitoferrin-1 and mitoferrin-2. Supporting these in vitro findings, their analysis of the TCGA database revealed that the expression of mitoferrin-1 correlated positively with the expression of key Warburg genes in patients, but in apparent contrast to the in vitro findings, the expression of mitoferrin-2 correlated negatively with the expression of the Warburg genes [33]. Li et al. also reported an association between the expression of both mitoferrins, mitochondrial iron and ROS levels, and the Warburg effect in pancreatic cancer cells, although the causal effect of the changes in mitoferrin expression was not examined. Interestingly, the expression of *SLC25A37* in patients with pancreatic cancer correlated negatively with patient survival, yet the expression of *SLC25A28* did not correlate with survival [48].

**Table 3:** Alteration of mitoferrin in different pathophysiological conditions.

Alteration in Mitoferrin	Associated Diseases	Type of Study (Used Model)	References
Mutation in mitoferrin-1	*Anemia*	In vitro	[46]
Dysregulated expression of mitoferrin	Myelodysplastic syndrome	In vitro	[62]
Loss of mitoferrin-1	Protoporphyria and hepatotoxicity	Animal (Mouse)	[25]
Mitoferrin-1 depletion	Erythropoietic protoporphyria	Patients’ tissue	[47]
Downregulation of mitoferrin-1 in hippocampus and peripheral blood	Major depressive disorder (MDD)	Patients’ tissue	[64]
Knockout of mitoferrin-1 in neuron	Impairment in spatial learning and memory	Animal (Mouse)	[65]
Overexpression of mitoferrin	Rescue of mitochondrial function and improvement in symptoms of Parkinson’s disease	*Drosophila melanogaster*	[66]
Overexpression of mitoferrin	Friedreich’s ataxia	*Drosophila melanogaster*	[67,68]
Downregulation of mitoferrin-1	Alzheimer’s disease	*Caenorhabditis elegans*	[69]
Increased expression of mitoferrin-1	Aging in adult skeletal muscle	Patients’ tissue	[70]
Knockout of mitoferrin-2	Reduced fertility	Mouse and *Drosophila melanogaster*	[71,72]
Loss of mitoferrin in hepatocytes	Disruption of liver regeneration	Animal (Mouse)	
Knockdown of mitoferrin-1 and mitoferrin-2	Impaired insulin sensitivity, suppress adipogenic differentiation	In vitro	[73]
Increased expression of mitoferrin-2	Atherosclerosis	Animal (Mouse)	[39]
Increased expression of mitoferrin-2	Huntington’s disease	Animal (Mouse)	[74]
Upregulation of mitoferrin-2	Head and neck cancer	In vitro	[75]
Downregulation of mitoferrin-2	Hepatocellular carcinoma	In vitro	[32]
Increased expression of mitoferrin-1	Pancreatic tumorigenesis	Animal (Mouse)	[48]

## 6. Conclusions

As the sole importers of iron into the mitochondria, the mitoferrin proteins are essential for maintaining the mitochondrial and cellular iron and redox homeostasis in eukaryotic cells. As a result of this critical function, mitoferrins regulate a variety of fundamental cellular processes, and the dysregulation of these proteins has been found to underlie the development of many diseases and disorders. However, the unique mechanisms regulating the expression and function of each mitoferrin are not clearly understood. Further studies in this regard should promote a better understanding of the mechanisms driving the pathologic alterations in cellular and mitochondrial iron metabolism; this research could lead to a paradigm shift, identifying the mitoferrins as drivers of so many seemingly diverse diseases.

## Figures and Tables

**Table 1:** Comparative chromosomal location, Ensembl accession number, transcript ID, exon count, uniprot accession number, and protein length of human mitoferrin-1 and mitoferrin-2.

	Gene			mRNA Seq			Protein Sequence		
Name	Chromosomal Location	Ensembl Accession No.	Transcripts	Ensembl Transcript ID	Exon Count	Coding Exons	Uniprot Accession No.	Isoform	Protein Length (aa)
Mitoferrin-1	8p21.2	ENSG00000147454	12	ENST00000519973.6	9	4	Q9NYZ2	3	338
Mitoferrin-2	10q24.2	ENSG00000155287	4	ENST00000370495.6	11	4	Q96A46-1	3	364
